# The value of targeted CXCR4 ^18^F-AlF-NOTA-pentixafor PET/CT for subtyping primary aldosteronism

**DOI:** 10.3389/fendo.2025.1533295

**Published:** 2025-02-27

**Authors:** Yushi Peng, Fangansheng Chen, Rui Yao, Junping Lan, Yinuo Fu, Kaifeng Ye, Zhiqiang Wang, Qianxiu Zhao, Xiaowei Ji, Kang Xia, Guoqing Zhu, Kewen Zheng, Xuemei Gu, Kun Tang

**Affiliations:** ^1^ Department of Nuclear Medicine, The First Affiliated Hospital of Wenzhou Medical University, Wenzhou, China; ^2^ Department of Radiology, The First Affiliated Hospital of Wenzhou Medical University, Wenzhou, China; ^3^ The First School of Clinical Medicine, Wenzhou Medical University, Wenzhou, China; ^4^ Department of Endocrinology, The First Affiliated Hospital of Wenzhou Medical University, Wenzhou, China; ^5^ Department of Interventional Radiology, The First Affiliated Hospital of Wenzhou Medical University, Wenzhou, China; ^6^ Department of Urology, The First Affiliated Hospital of Wenzhou Medical University, Wenzhou, China; ^7^ Key Laboratory of Intelligent Treatment and Life Support for Critical Diseases of Zhejiang Province, Wenzhou, China; ^8^ Key Laboratory of Novel Nuclide Technologies on Precision Diagnosis and Treatment & Clinical Transformation of Wenzhou City, Wenzhou, China

**Keywords:** primary aldosteronism, CXCR4, 18 F-pentixafor, PET/CT, subtyping, treatment

## Abstract

**Objective:**

The study aimed to investigate the diagnostic value of ^18^F-AlF-NOTA-Pentixafor PET/CT in subtyping primary aldosteronism (PA).

**Methods:**

This study enrolled 88 patients with PA or nonfunctional adenoma (NFA) for ^18^F-Pentixafor PET/CT scan. Of these, 20 patients underwent adrenal venous sampling (AVS), and 65 underwent adrenalectomy and postoperative follow-up.

**Results:**

In 88 patients, 76 were diagnosed with unilateral PA (UPA), 4 were diagnosed with bilateral PA (BPA), and 8 were diagnosed with NFA, resulting in a total of 95 lesions. To identify UPA, visual analysis received a specificity of 94.12% and a sensitivity of 89.74%. The optimal cutoff values for SUV_max_ at 5.45, the lesion-to-normal adrenal ratio (LAR) at 1.43, and lesion-to-liver ratio (LLR) all yielded a specificity of 100% and a sensitivity of 79.49%, 83.33%, and 80.77%, respectively. In 15 adrenal lesions with similar uptake to contralateral and adjacent normal adrenal tissue (defined as warm lesions), 7 were confirmed as UPA, 4 were confirmed as BPA, and 4 were confirmed as NFA. Furthermore, among the 20 patients who underwent AVS, the concordance rate of AVS and PET/CT visual analysis for PA subtyping was 65.00%.

**Conclusions:**

The CXCR4-targeted ^18^F-AlF-NOTA-pentixafor PET/CT is a valuable noninvasive tool for diagnosing UPA, demonstrating high sensitivity and specificity. More attention should be paid to warm adrenal lesions for their high diagnostic ambiguity probability.

## 1 Introduction

Primary aldosteronism (PA), resulting from excessive aldosterone secretion by the adrenal cortex, is a clinical syndrome characterized by hypertension with or without hypokalemia (1). Studies have demonstrated that PA is a common syndrome that may be a primary contributor to hypertension pathogenesis, and the prevalence of PA reaches over 20% in individuals with resistant hypertension (2, 3). Compared to primary hypertension, PA is more likely to lead to serious complications, such as aortic coarctation, severe arrhythmias, stroke, and renal failure (4). Currently, the most effective treatment for PA is unilateral adrenalectomy, and patients who undergo this procedure have considerably greater biochemical and clinical remission rates than those who receive medications, with both immediate and long-term benefits (5). PA cases can be subtyped as unilateral primary aldosteronism (UPA) and bilateral primary aldosteronism (BPA). UPA includes cases of unilateral autonomous excessive aldosterone secretion without contralateral secretion, as well as cases of bilateral autonomous asymmetric excessive aldosterone secretion whose dominant side has a clear advantage in secretory activity compared to the contralateral gland. BPA refers to cases of bilateral autonomous symmetrical excessive aldosterone secretion (6–8). The first-line treatment for UPA is surgery, and medication therapy is the recommended treatment for BPA. Thus, PA subtyping is essential for the optimal treatment management of PA patients.

At the present stage, the subtyping diagnosis of PA is mainly based on adrenal imaging and adrenal venous cannulation (AVS) to determine the location and functionality of the lesion (9). Computed tomography (CT) is the first choice for adrenal imaging. However, small adenomas or nodules with a diameter of less than 1 cm are often missed. It is hard to determine functional laterality of PA and differentiate between functional and non-functional adrenal nodule, with an overall accuracy of 60% to 70% in the diagnosis of PA subtyping (10). AVS is considered to be the gold standard for PA subtyping, which can clarify the existence of unilateral dominant secretion. However, since AVS is invasive, costly, and technically challenging, and carries risks of intubation failure and postoperative complications, it is difficult to conduct this procedure on a large scale in hospitals of all levels.

Radionuclide functional imaging is considered a promising novel noninvasive method for PA subtyping and has been investigated in prior research. As the most extensively utilized radiotracer for functional imaging, ^18^F-FDG tracks glucose uptake and metabolism, predominantly in tumor imaging, but exhibits no significant uptake in aldosterone-secreting adenomas (11, 12). Metomidate is an inhibitor of aldosterone synthase (CYP11B2). Research suggests that ^11^C-metomidate (^11^C-MET) PET/CT imaging exhibits promising diagnostic efficacy for aldosterone-producing adenomas (APA), with sensitivities ranging from 55% to 76% and specificities from 44% to 87% (13–15). Despite these findings, the clinical application of ^11^C-MET and its fluorine-18 analogue is constrained by the requirement for pre-treatment with dexamethasone. This underscores the critical need for alternative diagnostic methodologies to enhance PA subtyping.

C-X-C chemokine receptor 4 (CXCR4), a typical G protein-coupled receptor that stimulates cell migration and activation upon activation, is highly expressed on APA cell membranes and is significantly correlated with aldosterone synthase (CYP11B2) expression (16, 17). Numerous recent studies have demonstrated the clinical value of CXCR4-targeted ^68^Ga-Pentixafor PET/CT in the subtyping diagnosis of PA. Nevertheless, the inherent limitations of ^68^Ga, such as its short half-life and the low scalability of ^68^Ge/^68^Ga generators, have hindered its clinical application. These limitations can be addressed through the usage of ^18^F-labeled alternatives (18). Currently, there are few reports, both domestically and internationally, on the synthesis of CXCR4-targeted ^18^F-Pentixafor PET/CT. Moreover, to the best of our knowledge, no research has been published on the application of ^18^F-Pentixafor for diagnosing PA.

Therefore, the aim of this study was to develop a robust synthesis method for the novel molecular probe ^18^F-AlF-NOTA-Pentixafor, evaluate its biodistribution, and, most importantly, investigate its clinical application potential.

## 2 Materials and methods

### 2.1 Synthesis of ^18^F-AlF-NOTA-pentixafor

**Figure 1:**
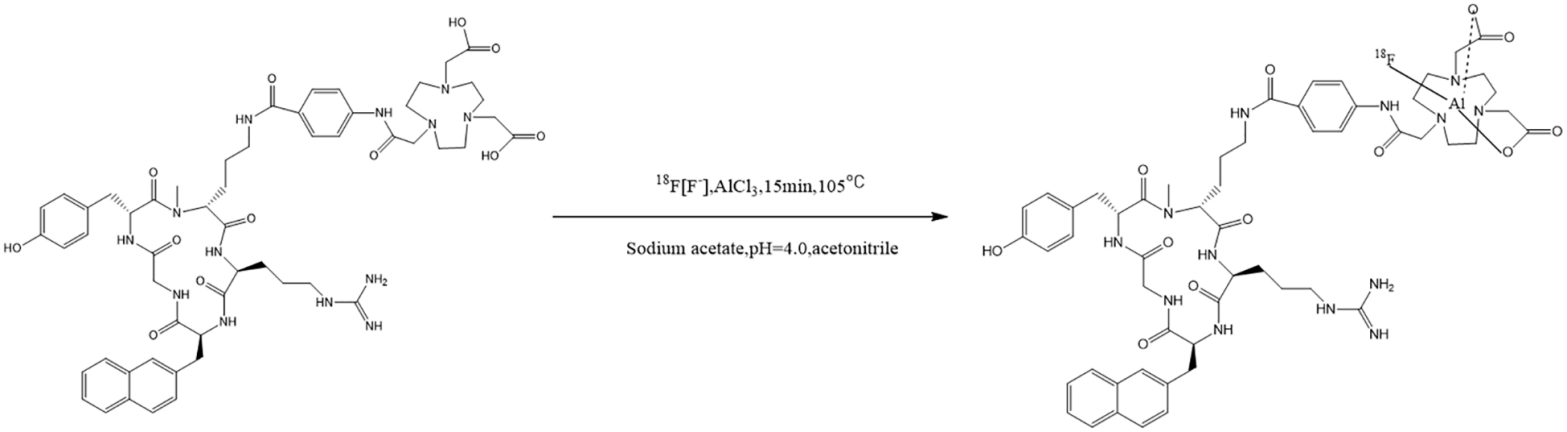
The synthetic chemical formula of ^18^F-AlF-NOTA-Pentixafor.

The ^18^F- isotope was produced by proton irradiation of an ^18^O-H_2_O target in the cyclotron and passed through a QMA column to capture the ^18^F-. The ^18^F- was then eluted with 0.5 mL of saline into the reaction tube, where a mixed solution containing 10 μL of aqueous AlCl_3_ (1 μg/mL), 200 μL of pH=4 acetate buffer, and 0.6 mL of precursor solution (200 μg in 0.6 mL acetonitrile) was added sequentially. The reaction mixture was heated to 105 ℃ for 15 min, and upon cooling to room temperature, it was transferred to an HLB column for purification. The mixture was rinsed with 20 mL of water for injection, eluted with 2 mL of 50% ethanol, and the final solution was filtered through a 0.22 μm sterile filter membrane. The final product was a colorless, transparent liquid with an ethanol content not exceeding 10% and a pH ranging from 5 to 8. The synthetic chemical formula is provided in **Figure 1**.

### 2.2 Study population

**Figure 2:**
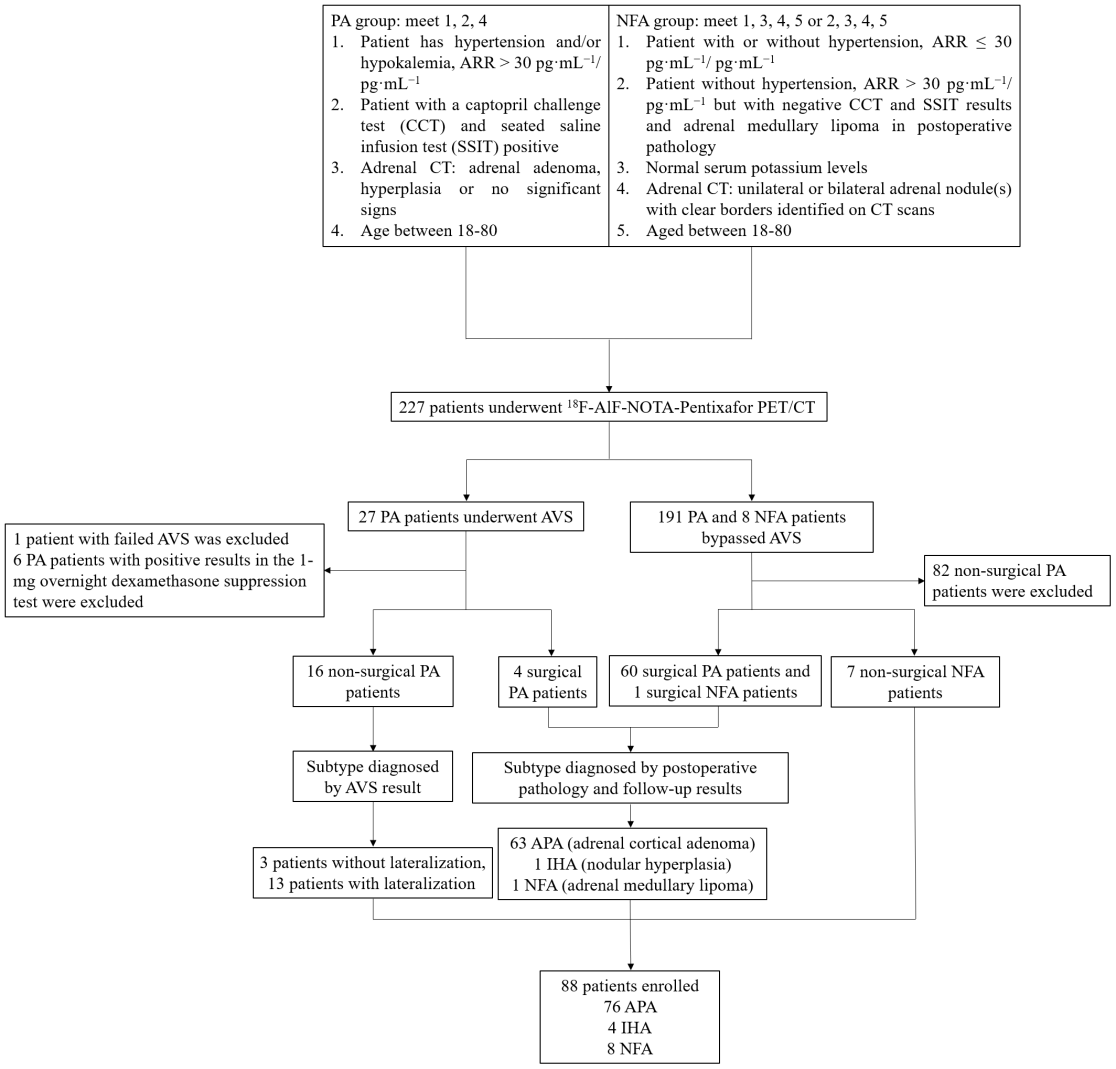
Patient inclusion flowchart.

Patients diagnosed with PA or NFA, aged 18-80 years, at the First Affiliated Hospital of Wenzhou Medical University between May 25^th^, 2023, and April 30^th^, 2024 were recruited for the study (**Figure 2**). All participants provided written informed consent, and the study protocol was approved by the Medical Ethics Committee of the First Affiliated Hospital of Wenzhou Medical University (Approval Number: KY2024-R245).

The diagnosis of PA was based on the clinical guidelines from the subcommittee of the Endocrine Society (1). All patients underwent PA screening using the plasma aldosterone–renin ratio (ARR) and received adrenal CT scans (slice thickness: 1.25 mm). Patients who tested positive in the screening (ARR > 30 pg·mL^−1^/pg·mL^−1^)) proceeded to confirmatory testing with a captopril challenge test (CCT) and seated saline infusion test (SSIT). All included patients underwent a 1-mg overnight dexamethasone suppression test (ODST) to exclude cortisol co-secretion in PA patients or mild autonomous cortisol secretion in NFA subjects. Patients who had unilateral or bilateral adrenal nodule(s) with clear borders identified on CT but presenting negative CCT and SSIT results or negative screening test results, along with normal serum potassium levels, were included as NFA. P The PA group was further subtyped into UPA and BPA. PA meeting any of the following criteria were classified as UPA: 1) unilateral lesion identified in AVS; 2) adrenal cortical adenoma confirmed by postoperative pathology, and complete or partial biochemical and clinical remission after surgery based on the Primary Aldosteronism Surgery Outcome criteria (19) (follow-up time: 1-6 months). PA meeting any of the following criteria were classified as BPA: 1) bilateral lesions identified in AVS; 2) adrenal cortical hyperplasia confirmed by postoperative pathology, and partial or no biochemical and clinical remission after surgery based on the Primary Aldosteronism Surgery Outcome criteria (follow-up time: 1-6 months) (20). Subtype diagnosis was determined based on postoperative pathological and follow-up results when two criteria were contradictory. All enrolled patients underwent 18F-AlF-NOTA-Pentixafor PET/CT imaging.

### 2.3 ^18^F-AlF-NOTA-Pentixafor PET/CT scanning and image analysis

Patients maintained a normal diet without any special preparation. ^18^F-AlF-NOTA-Pentixafor was administered intravenously at a dose of 0.1 mCi/kg. Approximately 60 minutes after the ^18^F-AlF-NOTA-Pentixafor injection, a local PET/CT scan of the upper abdomen was performed. Twenty-one patients diagnosed with or suspected of PA, who underwent 18F-AlF-NOTA-Pentixafor PET/CT scan between July 27^th^ and Oct. 19^th^ in 2023 at the first affiliated hospital of Wenzhou Medical University got additional PET/CT scan at 20 minutes post-injection (p.i.). Studies were performed on two dedicated PET/CT scanners (Gemini TF 64, Philips Medical Systems, Netherlands, and uMI Panorama 35S, equipped with third-generation TOF 3D acquisition technology) with the following parameters: 120 kV, 80 mA, pitch of 0.829, tube rotation time of 0.5 seconds per rotation, and reconstruction thickness and interval of 5.0 mm.

Two nuclear medicine physicians certified by the committee, who were blinded to the patient’s clinical information, evaluated the PET/CT data. Any inconsistencies in their evaluation were resolved through negotiation, and the final decision was reached through consensus. Lesions were considered positive (also referred to as hot lesions) on PET/CT based on visual analysis if the adrenal nodule(s) showed higher uptake than the normal ipsilateral and contralateral adrenal glands. Adrenal lesions with similar uptake to the contralateral and adjacent normal adrenal tissue on visual assessment were classified as warm lesions. Adrenal lesions with lower uptake than the contralateral and adjacent normal adrenal tissue on visual assessment were classified as cold lesions. Warm and cold lesions were collectively referred to as negative lesions. The main lesion was identified as the adrenal nodule with the highest ^18^F-AlF-NOTA-Pentixafor uptake and clear borders in patients with multifocal nodules. Lesions identified on CT, or those with no abnormalities on CT but suspected of increased tracer uptake on PET, were designated as regions of interest, and the maximal standardized uptake value (SUV_max_) was measured in these regions. The liver SUV_max_ was defined as the average SUV_max_ of five round spheres, each with a diameter of 2 cm, selected from the liver. Specific uptake value ratios, such as the lesion-to-liver ratio (LLR) and the lesion-to-normal adrenal ratio (LAR), were calculated. Additionally, in 18 patients who underwent AVS, SUV_max_ values within each adrenal gland were recorded. The side with the higher SUV_max_ in both adrenal glands was considered the dominant side. The lateralization index (LI) based on SUV_max_ was calculated as (SUV_max_ of dominant side)/(SUV_max_ of non-dominant side).

### 2.4 Adrenal venous sampling interpretation

Patients diagnosed with PA underwent AVS within 3 months of completing ^18^F-pentixafor PET/CT to determine the lateralization of aldosterone hypersecretion. The decision on whether to perform AVS was made by endocrinologists with over 20 years of experience based on the clinical indicators, PET/CT results, and the patient’s inclination. AVS without adrenocorticotropic hormone stimulation was performed in the morning between 8:00 AM and 12:00 PM. Successful catheterization is considered if the adrenal cortical cortisol/peripheral venous cortisol ratio (ie, selectivity index) is 2 or greater.

A diagnosis of UPA was made if LI based on AVS was 4 or greater or 2 to 4 in combination with contralateral suppression or CT showing a typical adenoma on the dominant side, while those with LI based on AVS of less than 2 or 2 to 4 without meeting the previously described criteria were diagnosed as BPA (21, 22).

### 2.5 Pathologic diagnosis and follow-ups

The pathological results of all surgical patients were independently assessed by a pathologist who was blinded to the clinical and imaging findings.

At least one follow-up was conducted by endocrinologists within 1 to 6 months after surgery (routine follow-up was scheduled at 1, 3, and 6 months post-surgery). For patients with PA, the Postoperative Aldosterone Outcome Score (PASO) system was used to evaluate their outcomes, categorizing patients into three groups: (1) Cure: blood pressure is normal without the use of antihypertensive medication; normal serum potassium and ARR, or ARR remains elevated but aldosterone levels can be suppressed in confirmatory testing; (2) Improvement: blood pressure has decreased to normal or has remained relatively unchanged with a reduction in antihypertensive medication use; normal serum potassium levels, elevated ARR, but plasma aldosterone levels have decreased by 50% compared to pre-surgery levels, or confirmatory test results have improved compared to baseline; (3) No improvement: antihypertensive medication dosage remains the same or has increased, and blood pressure has not improved or has worsened; low serum potassium and/or elevated ARR and/or aldosterone levels are not suppressed in confirmatory testing (19).

### 2.6 Statistical methods

IBM SPSS Statistics 25.0 was used for statistical analysis. The Kolmogorov-Smirnov (K-S) test was employed to assess normality initially. Normally distributed data were reported as mean ± SD (χ ± S), and non-normally distributed data were reported as median (P_25_, P_75_). Nonparametric tests were used for between-group comparisons. Categorical data were presented as frequencies (percentages), and between-group comparisons were conducted using χ² or Fisher’s exact test when appropriate. Correlation analysis was performed using Pearson or Spearman correlation analysis. The diagnostic value of SUV_max_, LLR, LAR, and LI of ^18^F-AlF-NOTA-pentixafor PET/CT was evaluated using receiver operating characteristic (ROC) curve analysis. Data visualization was conducted using GraphPad Prism 9.3 software and Origin 2022. This study employed a 2-tailed test, and a P value less than 0.05 was considered statistically significant for all tests.

## 3 Results

### 3.1 Tracer radiochemistry and biodistribution

^18^F-AlF-NOTA-pentixafor was synthesized with radiochemical yields of (37 ± 8.5) %. Radiochemical purities were > 99.5% as confirmed by radio-TLC.

**Figure 3:**
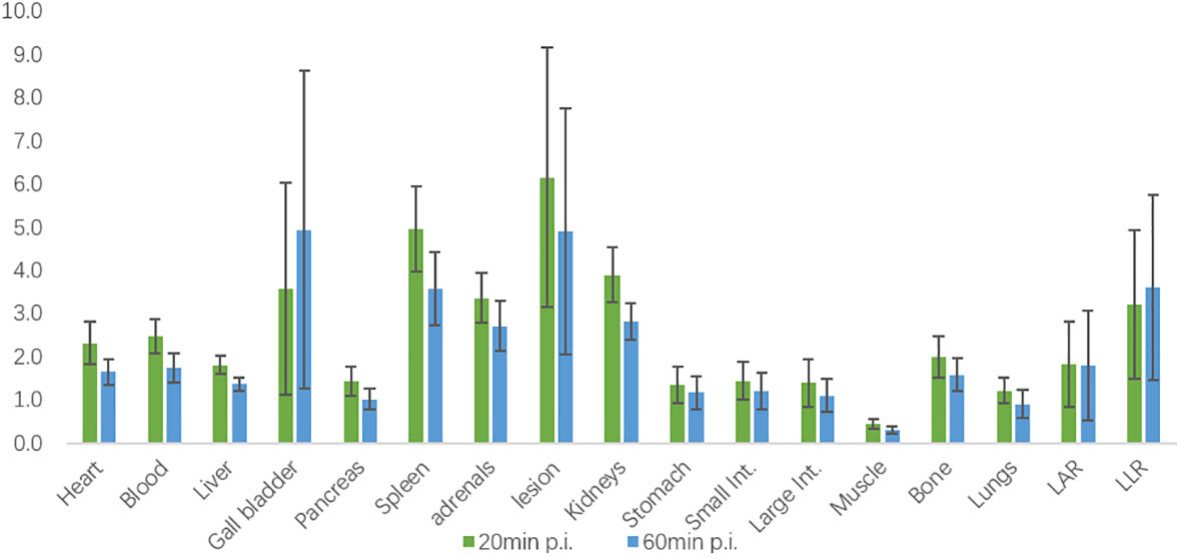
Biodistribution of ^18^F-AlF-NOTA-pentixafor in 21 patients diagnosed or suspected of PA at 20 minutes and 60 minutes post-injection (p.i.) measured by SUV_mean_.

**Figure 4:**
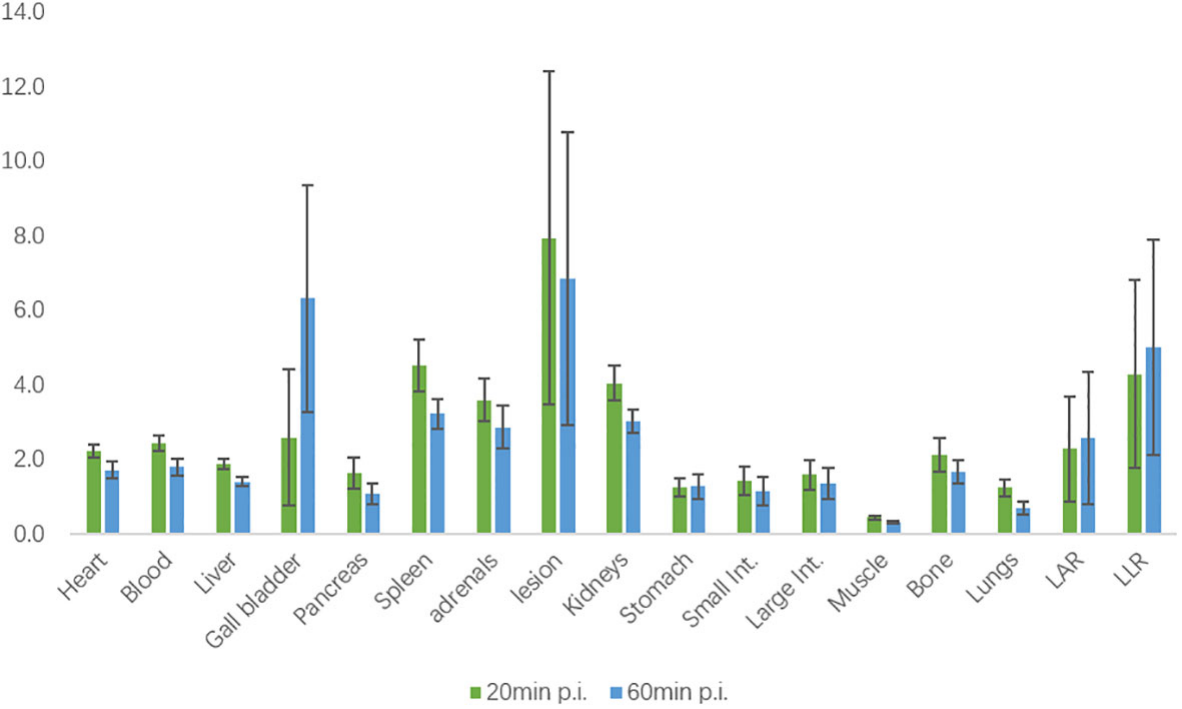
Biodistribution of ^18^F-AlF-NOTA-pentixafor in 6 UPA patients at 20 minutes and 60 minutes post-injection (p.i.) measured by SUV_mean_.

The biodistribution of ^18^F-AlF-NOTA-pentixafor in 21 patients diagnosed with or suspected of PA at 20 minutes and 60 minutes post-injection (p.i.) is shown in **Figure 3**. Among these patients, six patients were later diagnosed with UPA, and one patient was diagnosed with BPA. All the organs in the upper abdomen (including adrenal gland lesions) exhibited higher mean SUV_mean_ at 20 minutes p.i. than it at 60 minutes p.i., except for gallbladder. Specifically, the spleen exhibited the highest uptake at 20 minutes p.i., while the gallbladder exhibited the highest uptake at 60 minutes p.i. The adrenal gland lesion exhibited the highest SUV_mean_ in the upper abdomen. In 6 UPA patients, LAR and LLR at 60 minutes p.i. (2.57[95%CI, 0.71-4.43] and 5.00[95%CI, 1.96-8.04]) was higher than LAR and LLR at 20 minutes p.i. (2.28[95%CI, 0.80-3.77] and 4.28[95%CI, 1.64-6.92]) but with no statistical differences (P=0.766 and 0.657, respectively) (**Figure 4**).

### 3.2 Clinical characteristics and SUV_max_ of included patients and their correlation

**Figure 5:**
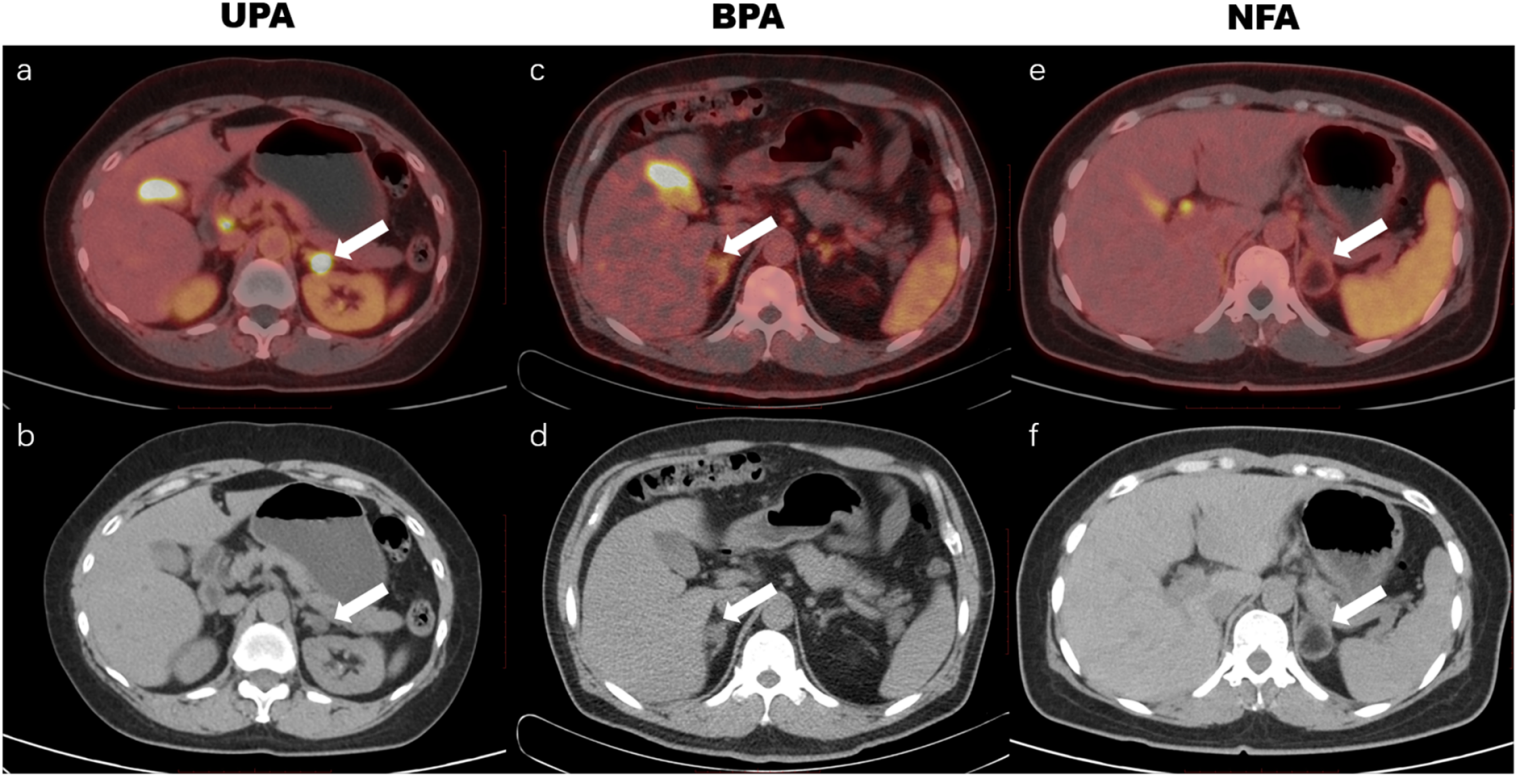
^18^F-AlF-NOTA-pentixafor PET/CT imaging of patients with unilateral primary aldosteronism (UPA), bilateral primary aldosteronism (BPA), and nonfunctional adenoma (NFA). The UPA patient was a 61-year-old woman with hypertension for 10 years and hypokalemia for 2 years, with maximum blood pressure of 163/105 mm Hg, and minimum blood potassium of 2.69 mmol/L. A positive uptake (SUV_max_ of 31.5, white arrow in a, b) was observed in the left adrenal nodule, and postoperative pathology revealed a left adrenal cortical adenoma. The BPA patient was a 74-year-old man with hypertension for 13 years and hypokalemia, maximum blood pressure of 160/99 mm Hg, and minimum blood potassium of 3.05 mmol/L. ^18^F-AlF-NOTA-pentixafor PET/CT showed a warm lesion in the right adrenal (white arrow in c, d) with an SUV_max_ of 4.0. The lateralization index (LI) based on adrenal venous sampling (AVS) was 1.3, which indicated bilateral lesions. The NFA patient was a 46-year-old woman with an incidental adrenal nodule and aldosterone/renin ratio of 138.5, without hypertension or hypokalemia. The ^18^F-AlF-NOTA-pentixafor PET/CT exhibited a cold lesion in the left adrenal (white arrow in e, f) with an SUV_max_ of 2.9, and postoperative pathology revealed a left adrenal medullary lipoma.

Overall, there were 45 men and 43 women in our cohort (mean age, 53.52 ± 10.28 years). Among the 88 included patients, 20 underwent AVS and 65 underwent adrenalectomy. Based on clinical evaluation, pathology, and follow-up data, 76 cases were diagnosed as UPA, 4 cases as BPA, and 8 cases as NFA (UPA lesions were classified as surgically eligible lesions, BPA and NFA lesions were classified as surgically ineligible lesions). No significant demographic differences (including age, gender, and BMI) were observed among the three groups. Compared with the UPA group, the BPA group exhibited statistically lower SUVmax, while the NFA group demonstrated statistically higher serum potassium and plasma renin concentration (PRC), and lower plasma aldosterone concentration (PAC), ARR, and SUVmax (**Table 1**, **Figure 5**). No significant statistical differences were observed in semi-quantitative parameters, including SUV_max_, LAR, and LLR, between the BPA group and the NFA group (as shown in **Table 1**).

**Table 1:** Clinical characteristics and maximum standardized uptake values of included 88 patients.

Characteristic	Patients, Median (P_25_, P_75_)				P
	Total	UPA	BPA	NFA	
Age, y, mean ± SD	53.52 ± 10.28	53.13 ± 10.38	53.75 ± 14.52	57.13 ± 7.28	.690
Gender (male/female)	45/31	39/37	2/2	4/4	1.000
BMI, mean ± SD	25.25 ± 3.24	25.15 ± 3.31	22.95 ± 2.39	27.16 ± 1.84	.378
Number of hypertension	85/88	74/76	4/4	7/8	.359
Duration of hypertension, y	7.00 (2.00,13.50)	8.00 (3.00,20.00)	3.50 (1.25,11.00)	3.00 (0.38,6.50)	.039
Systolic pressure, mmHg	162.00 (150.00,179.00)	163 (152,179)	155.50 (134.25,162.50)	143.00 (139.25,171.00)	.033
Diastolic pressure, mmHg	96 (89,108)	99 (89,108)	92.50 (89.75,108.00)	94.00 (81.25,104.00)	.509
Number of hypokalemia	66/88	64/76	2/4	0/8	<.001
Serum potassium, mmol/L	3.20 (2.90,3.49)	3.12 (2.83,3.34)	3.55 (3.14,3.79)	3.98 (3.91,4.05)^a^	<.001
Plasma aldosterone concentration, pg/mL	340.70 (195.49,541.58)	332.30 (235.49,625.66)	215.25 (159.22,301.51)	161.16 (142.99,177.46)^a^	<.001
Plasma renin concentration, pg/mL	2.05 (1.01, 5.28)	2.02 (0.90, 4.38)	1.41 (0.71, 2.92)	8.23 (2.13, 17.60)^a^	.029
ARR, pg·mL^−1^/pg·mL^−1^	158.30 (65.87,393.96)	188.56 (68.37,462.00)	170.25 (93.97,266.09)	23.28 (8.85,79.12)^a^	.002
Lesion diameter, mm	14.00 (11.00,18.00)	13.00 (11.00,18.00)	11.00 (8.50,17.25)	20.50 (14.75,26.50)	.041
SUV_max_	8.35 (4.35,16.00)	10.00 (5.70,17.10)	3.90 (3.28,4.83)^a^	2.90 (2.58,3.65)^a^	<.001
LAR	2.17 (1.17,4.54)	2.97 (1.56,4.99)	1.11 (1.07,1.26)	0.83 (0.77,0.94)^a^	<.001
LLR	4.58 (2.13,10.10)	6.47 (3.43,10.45)	2.55 (2.15,2.83)	2.05 (1.55,2.10)^a^	.001

Compared with the UPA group, ^a^P is less than 0.05.

**Figure 6:**
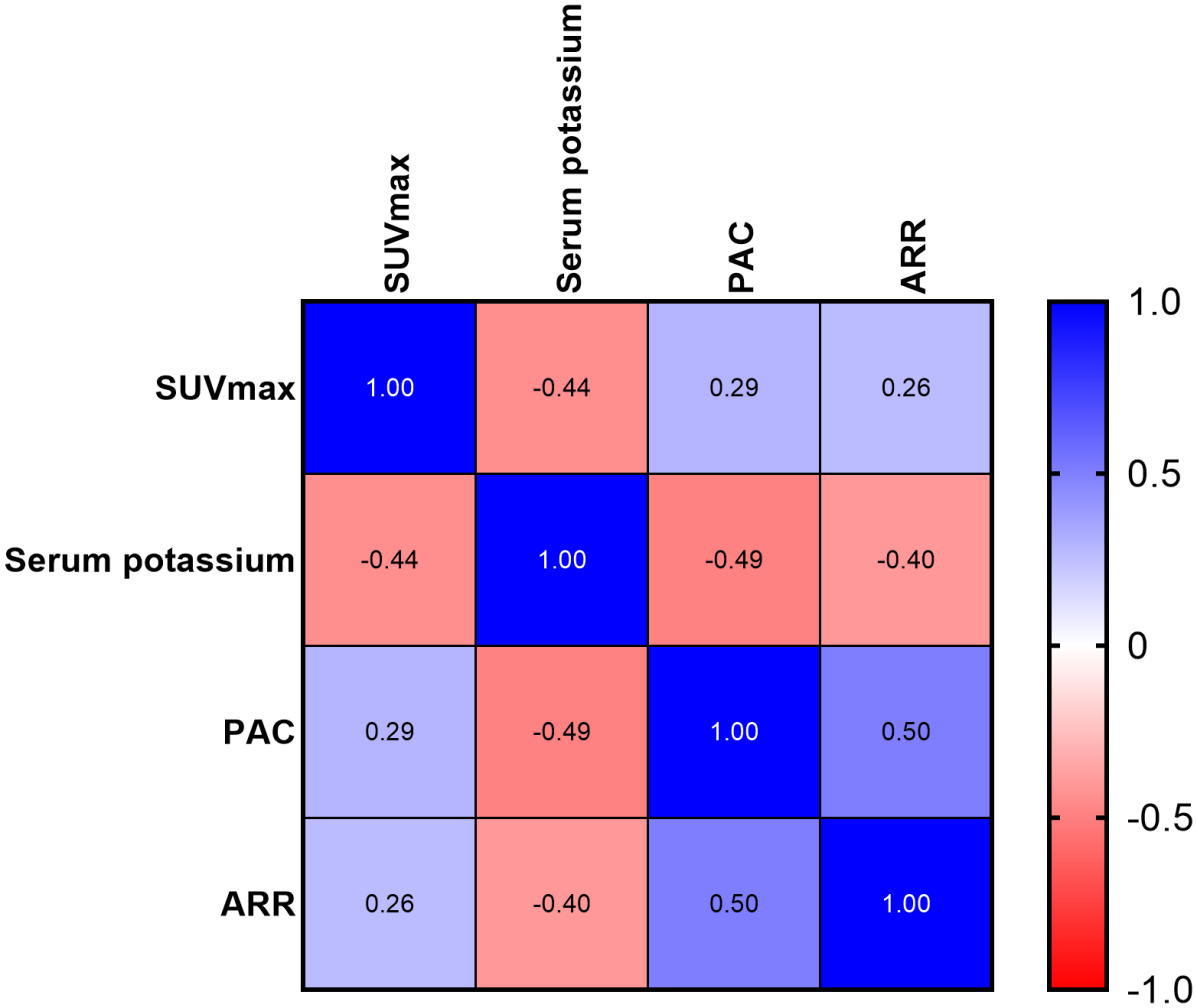
Correlation heatmap of SUV_max_, serum potassium, PAC, and ARR (all proved P value < 0.05).

Besides, we conducted a correlation analysis among SUV_max_ and clinical characteristics. The results showed that SUV_max_ was negatively correlated with blood potassium concentration (r = − 0.444; P < 0.001) and age (r = − 0.334; P = 0.001), and positively correlated with PAC (r = 0.287; P = 0.007) and ARR (r = 0.265; P = 0.013) (partly shown in **Figure 6**). There was no correlation of SUV_max_ with sex, BMI, duration of hypertension, systolic blood pressure, diastolic blood pressure, or lesion diameter. In PA patients, a significant correlation was observed between SUV_max_ and lesion diameter (r = 0.351; P = 0.001).

### 3.3 Diagnostic Accuracy of ^18^F-AlF-NOTA-pentixafor PET/CT for surgically eligible lesions

In 88 patients, 95 lesions exhibited abnormalities in PET or CT scans (comprising 78 cases of surgically eligible lesions and 17 cases of surgically ineligible lesions). A total of 78 UPA and 4 BPA lesions were confirmed by AVS and/or postoperative pathology, while the remaining 13 NFA lesions were confirmed through clinical evaluation and/or postoperative follow-up evidence. Among the 95 lesions, 71/78 surgically eligible lesions demonstrated positive findings (sensitivity 89.74%), and 16/17 surgically ineligible lesions showed negative findings (specificity 94.12%) in visual analysis of ^18^F-AlF-NOTA-pentixafor PET/CT. The median SUV_max_, LAR, and LLR of the surgically eligible lesions were 9.85(5.70,16.90), 2.85(1.66,4.52), and 5.90(3.59,9.75), respectively, significantly higher than those of the surgically ineligible lesions of 3.40(2.90,3.90), 0.92(0.83,1.11), 2.06(1.71,2.38).

**Figure 7:**
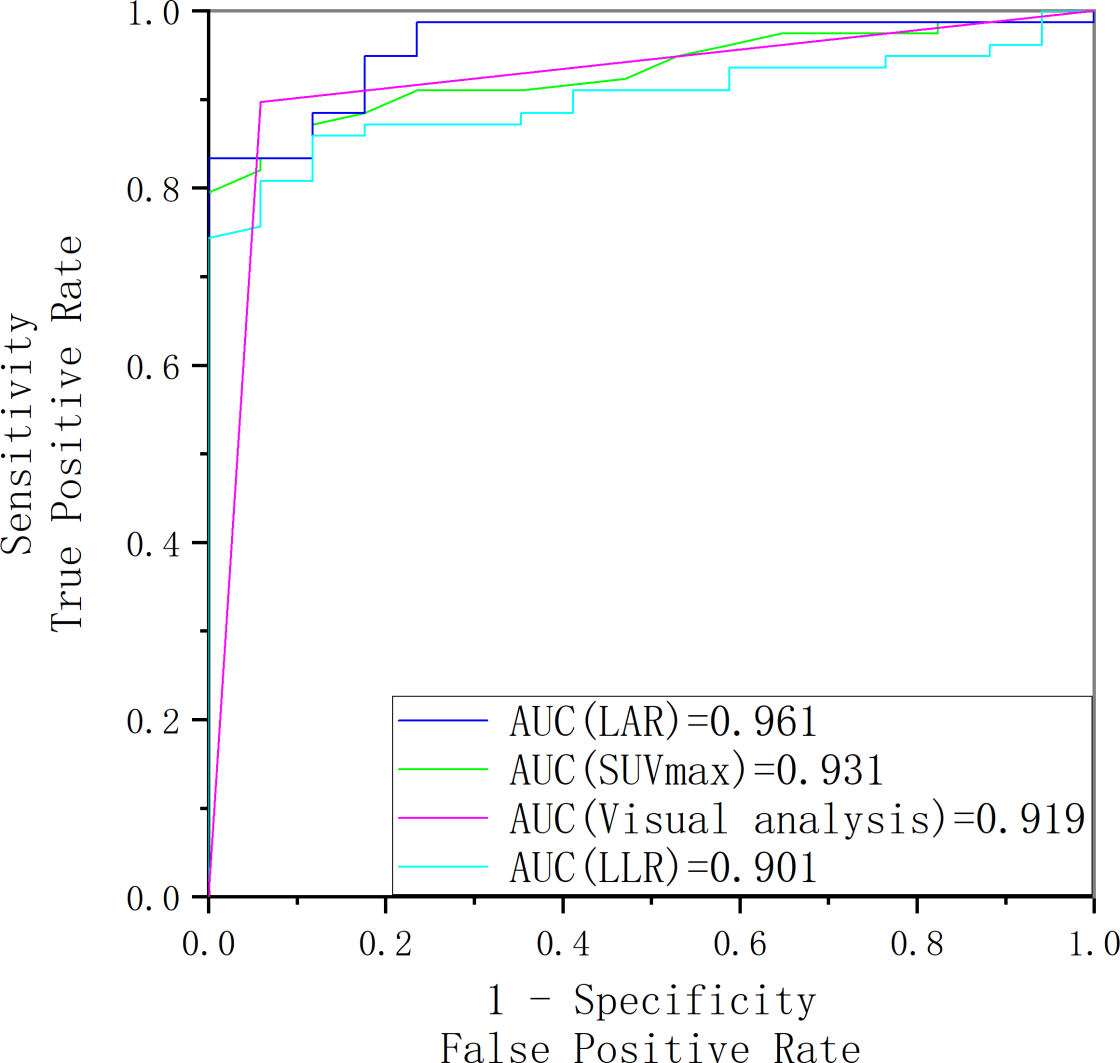
The receiver operating characteristic curve of SUV_max_, LAR, LLR and visual analysis for identifying surgically eligible lesions and surgically ineligible lesions.

**Figure 7** showed LAR based on ^18^F-pentixafor SUV_max_ had a higher AUROC (0.961 [95%CI, 0.899-1.023]) than SUV_max_ of the lesion (0.931 [95% CI, 0.841-1.021]), visual analysis (0.919 [95%CI, 0.830-1.009]), and LLR based on SUV_max_ (0.901 [95% CI, 0.785-1.016]) to diagnose surgically eligible lesions. Thereinto, the AUROC of LAR was statistically higher than that of LLR (P=0.008). To achieve the maximized Youden index, the optimal cutoff LAR, SUV_max_, and LLR was 1.43, 5.45, and 3.20, respectively, with a sensitivity of 83.33%, 79.49%, and 80.77%. No patients with surgically ineligible lesions would be misdiagnosed as surgically eligible lesions using these cutoffs with specificity of 100% in all (shown in **Table 2**).

**Table 2:** Visual and semi-quantitative analysis of ^18^F-AlF-NOTA-pentixafor PET/CT in surgically eligible lesions and surgically ineligible lesions.

	Total (n=95)	Surgically eligible lesions (n=78)	Surgically ineligible lesions (n=17)	P
Visual analysis				
Positive lesions (%)	71	70(89.74)	1 (5.88)	<.001
Negative lesions (%)	24	8(10.26)	16 (94.12)	
Semi-quantitative analysis				
SUV_max_ (≥5.45) (%)	62	62 (79.49)	0 (0)	<.001
SUV_max_ (<5.45) (%)	33	16 (20.51)	17 (100)	
Median SUV_max_ (Q25-Q75)	7.55 (3.90,13.60)	9.85 (5.70,16.90)	3.40 (2.90,3.90)	<.001
LAR (≥1.43) (%)	65	65 (83.33)	0 (0)	<.001
LAR (<1.43) (%)	30	13 (16.67)	17 (100)	
Median LAR (Q25-Q75)	2.09 (1.18,4.05)	2.85 (1.66,4.52)	0.92 (0.83,1.11)	<.001
LLR (≥3.20) (%)	64	63 (80.77)	1 (0)	<.001
LLR (<3.20) (%)	31	15 (19.23)	16 (100)	
Median LLR (Q25-Q75)	4.41 (2.14,7.98)	5.90 (3.59,9.75)	2.06 (1.71,2.38)	<.001

### 3.4 Effectiveness of ^18^F-AlF-NOTA-pentixafor PET/CT in detecting lesions with diameter 1 cm and ≥ 1 cm

To further evaluate the diagnostic value of ^18^F-AlF-NOTA-pentixafor PET/CT for lesions with different diameters, we divided 95 lesions into two groups: lesions with diameter < 1 cm and ≥ 1 cm. Among 95 lesions, 13 lesions examined with PET/CT were < 1 cm (10 nodules, 1 nodular thickening, 1 diffuse thickening, and 1 positive lesion without morphological abnormalities), while 82 were ≥ 1 cm in diameter (76 nodules and 6 nodular thickening). The diagnostic performance of ^18^F-AlF-NOTA-pentixafor PET/CT in identifying UPA was further analyzed in these 2 groups of patients and no significant demographical or biochemical differences were observed between the groups (**Supplementary Table 1**). The small lesion group (< 1 cm in diameter) has higher optimal cutoffs of metabolic parameters (SUV_max_=5.75, LAR=1.43, LLR=3.65) than the big lesion group (≥ 1 cm in diameter) (SUV_max_=5.15, LAR=1.47, LLR=3.20). Compared to the small lesion group, the large lesion group demonstrated higher sensitivity, specificity, and accuracy either in visual analysis or semi-quantitative parameters (**Table 3**). For lesions < 1 cm in diameter, LAR of 1.43 identified 9 UPA out of 11 lesions with an accuracy of 84.62% (sensitivity of 81.82%, specificity of 100%) (**Table 3**). For lesions ≥ 1 cm in diameter, visual analysis identified 61 UPA out of 67 lesions, with an accuracy of 92.68% (sensitivity of 91.04%, specificity of 100%) for identifying UPA.

**Table 3:** Diagnostic efficacy of ^18^F-AlF-NOTA-pentixafor PET/CT for lesions < 1 cm and ≥ 1 cm in diameter based on visual and semiquantitative analysis.

	True positive	True negative	False positive	False negative	Sensitivity	Specificity	Positive predictive value	Negative predictive value	Accuracy
Lesion <1 cm in diameter									
Visual analysis	8	1	1	3	72.73%	50%	88.89%	25%	69.23%
SUV_max_=5.75	8	2	0	3	72.73%	100%	100%	40%	76.92%
LAR=1.43	9	2	0	2	81.82%	100%	100%	50%	84.62%
LLR=3.65	7	2	0	4	63.64%	100%	100%	33.33%	69.23%
Lesion ≥1 cm in diameter									
Visual analysis	61	15	0	6	91.04%	100%	100%	71.42%	92.68%
SUV_max_=5.15	56	15	0	11	83.58%	100%	100%	57.69%	86.59%
LAR=1.47	56	15	0	11	83.58%	100%	100%	57.69%	86.59%
LLR=3.20	55	15	0	12	82.09%	100%	100%	55.56%	85.37%

### 3.5 Further analysis of patients misdiagnosed by ^18^F-AlF-NOTA-pentixafor PET/CT visual analysis

Since 7 out of 76 UPA patients showed negative findings in visual analysis, we decided to analyze the misdiagnosed reason further. All 7 missing UPA were warm lesions, with patients’ clinical and biochemical characteristics shown in **Table 4**.

**Table 4:** Clinical and biochemical characteristics of patients misdiagnosed by ^18^F-AlF-NOTA-Pentixafor PET/CT visual analysis.

Patient	Age, y	Sex	Serum potassium, mmol/L	PAC, pg/mL	PRC, pg/mL	ARR, pg·mL^−1^/pg·mL^−1^	Lesion diameter, mm	Characteristic of nodule	Lesion SUV_max_	MainLAR	LI based on AVS
No. 7	76	F	4.40	208.15	1.02	204.07	8	Unilateral single warm nodule	3.1	1.15	5.26
No. 11	58	M	3.70	733.34	0.5	14666.8	16	Bilateral negative nodules (left warm nodule and right cold nodule)	3.5	1.17	3.50
No. 13	54	M	3.55	252.32	5.26	47.97	7	Left multifocal warm nodules and right adrenal ramus medialis with increased uptake but no anatomical abnormalities	3.9	1.03	12.34
No. 21	56	M	3.49	255.12	12.67	20.14	10	Unilateral single warm nodule	4.2	1.27	6.14
No. 24	67	M	2.76	1822	11.51	158.3	13	Bilateral warm nodules	2.7	1.04	28.00
No.68	52	M	2.86	322.3	0.79	407.97	24	Bilateral warm nodules	3.4	1.31	2.00
No.96	53	F	3.03	123.66	1.37	90.26	8	Unilateral single warm nodule	3.9	1.44	N/A

All 69 positive main lesions (hot lesions) out of 88 patients were proved UPA while all 4 cold main lesions were proved NFA in clinical. In 15 warm lesions, 7 lesions were proved UPA, 4 lesions were proved BPA, and 4 were proved NFA. There was a high correlation between the final diagnosis and the degree of the main lesion uptake (χ2 = 48.502; P < 0.001) (**Table 5**).

**Table 5:** Correlation between ^18^F-AlF-NOTA-pentixafor PET/CT and final diagnosis.

^18^F-AlF-NOTA-Pentixafor	UPA (n=76)	BPA (n=4)	NFA (n=8)	P value
Positive lesions (%) (Hot lesions)	69(90.79)	0	0	χ2 = 48.502 P<0.001
Negative lesions (%)	7(9.21)	4 (100.00)	8 (100.00)	
Warm lesions (%)	7(9.21)	4 (100.00)	4 (50.00)	
Cold lesions (%)	0	0	4 (50.00)	

### 3.6 Concordance of ^18^F-AlF-NOTA-pentixafor PET/CT and AVS in patients with PA

**Figure 8:**
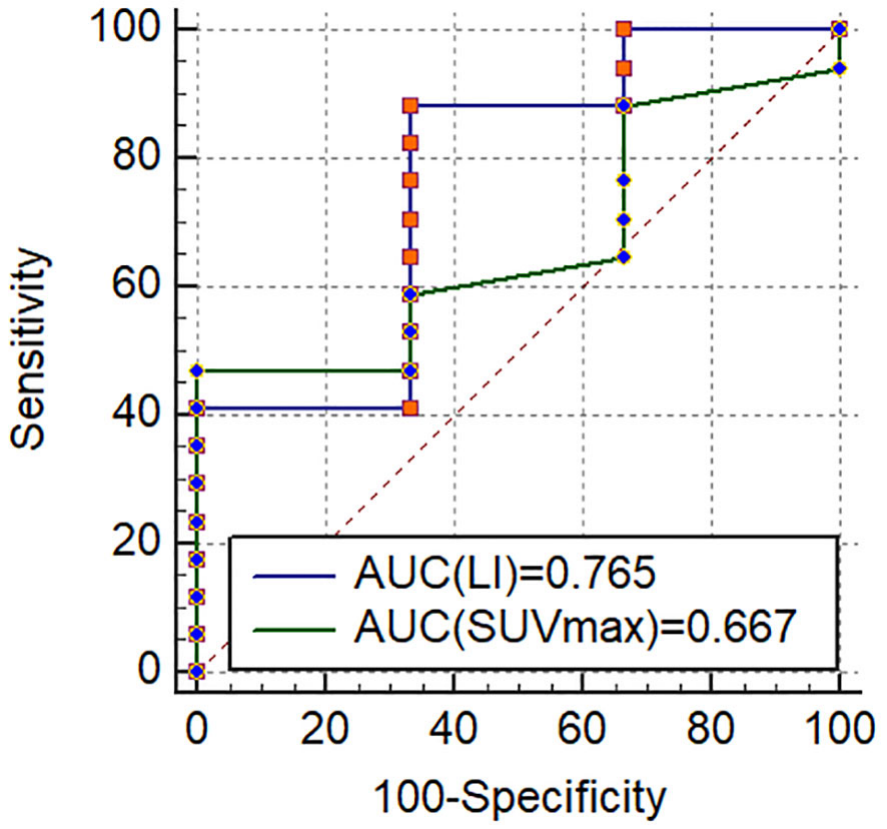
The receiver operating characteristic curves for diagnosis of UPA in 20 patients who underwent AVS.

Considering that the ultimate subtyping diagnosis needs to specify the hypersecretion side of the adrenal gland rather than simply distinguishing between unilateral or bilateral cases, we further conduct an analysis of the concordance rate of AVS and PET/CT visual diagnosis in 20 patients who underwent AVS (**Table 6**). Of 11 UPA patients identified by PET/CT visual analysis, 10 were confirmed as having ipsilateral dominant secretion by AVS. The single patient with non-matching laterality between AVS and PET/CT showed multifocal warm nodules on the dominant secretion side of AVS, while the contralateral adrenal ramus medialis showed increased uptake but no anatomical abnormalities. Additionally, 3 of 9 patients with BPA identified by PET/CT were confirmed as having BPA by AVS. Hence, the total concordance rate of AVS and PET/CT visual diagnosis was 65.00% (13/20). The clinical characteristics of 7 patients with a disagreement between PET/CT visual diagnosis and AVS were shown in **Supplementary Table 2**. The ROC analysis in the 20 patients showed that the area under the curve (AUC) for SUV_max_ and LI based on SUV_max_ reached 0.667 and 0.765, respectively, with the optimal cutoff values of SUV_max_=5.15 and LI=1.12 (**Figure 8**). However, employing SUV_max_=5.15 and LI based on SUV_max_=1.12 for subtyping diagnosis did not result in an improvement in the total concordance rates (65.00% (13/20); 55.00% (11/20)) when compared to visual analysis.

**Table 6:** Concordance of ^18^F-AlF-NOTA-pentixafor PET/CT and AVS in patients with PA.

Functional lateralization diagnosis based on AVS	Functional lateralization diagnosis based on ^18^F-Pentixafor PET/CT								
	Visual analysis			LI (cutoff=1.12)			SUV_max_ (cutoff=5.15)		
	Left (n=5)	Right (n=6)	Bilateral (n=9)	Left (n=10)	Right (n=6)	Bilateral (n=4)	Left (n=3)	Right (n=5)	Bilateral (n=12)
Left (n=7)	4	0	3	6	1	0	3	0	4
Right (n=10)	1	6	3	3	5	2	0	5	5
Bilateral (n=3)	0	0	3	1	0	2	0	0	3
**Concordance rate between AVS and PET/CT, % (n)**	65.00% (13/20)			65.00% (13/20)			55.00% (11/20)		

## 4 Discussion

^68^Ga-Pentixafor is the predominant CXCR4 imaging radiotracer, but it faces practical, regulatory, and economic barriers associated with ^68^Ge/^68^Ga-generators ^(^ 23, 24). These concerns could potentially be resolved through the introduction of a fluorine-18-labeled alternative with high production yield, enabling centralized production and distribution to remote PET centers. In our study, we successfully synthesized ^18^F-AlF-NOTA-pentixafor and for the first time applied ^18^F-pentixafor to clinical practice. The results indicated ^18^F-pentixafor PET/CT, as a non-invasive examination, could play an important role in the subtyping diagnosis of PA.

Our study demonstrates that ^18^F-pentixafor uptake in adrenal gland lesions of patients diagnosed or suspected of PA is higher than in any other organ in the upper abdomen, with excellent adrenal gland lesion/background ratios (LAR and LLR) for ^18^F-AlF-NOTA-pentixafor. As we did not synthesize ^68^Ga-pentixafor in our hospital, we roughly compared our biodistribution data of ^18^F-AlF-NOTA-pentixafor with that of ^68^Ga-NOTA-pentixafor in other studies and found similar biodistribution and high variability of the gall bladder in both radiotracers (25). Previous research by Hu et all (26) has suggested SUV_max_ of early ^68^Ga-pentixafor PET imaging may be better than SUV_max_ of delayed imaging. Yet in our study, LAR and LLR at 60 minutes p.i. were relatively higher than those at 20 minutes p.i. in 6 UPA patients with no statistical differences, which might be attributable to the small data set. Therefore, further validation with larger sample sizes is needed to determine the optimal time for performing ^18^F-pentixafor PET/CT scan.

Several previous studies have underscored the significance of targeted CXCR4 PET/CT in subtyping the diagnosis of primary aldosteronism (PA); however, their focus was solely on ^68^Ga-pentixafor. It is essential to explore the clinical applicability of the promising targeted CXCR4 radiotracer, ^18^F-pentixafor. In our study, SUV_max_s of the BPA group and NFA group were statistically lower than those of the UPA group. Furthermore, ^18^F-AlF-NOTA-pentixafor proved highly accurate in distinguishing surgically eligible lesions from those in the surgically ineligible lesions. The results indicated that 89.74% of surgically eligible lesions showed positive uptake in PET/CT, while 94.12% of surgically ineligible lesions (including BPA and NFA lesions) exhibited similar or lower contralateral and adjacent normal adrenal tissue uptake in PET/CT. In comparison to visual analysis, the optimal cutoff values of the semi-quantitative parameters including SUV_max_ (5.45), LAR (1.43), and LLR (3.20) show lower sensitivity (79.49, 83.33, 80.77) and higher specificity (100% in all cases). These results were consistent with the study of Zheng et al, which included 66 APA, 33 IHA, and 21 NFA patients to get ^68^Ga-pentixafor PET/CT scan (20). It is worth mentioning that, although Xiangya Hospital classified PA patients as APA and IHA in this study, these are substantially equivalent to UPA and BPA. However, the optimal cutoff SUV_max_ (5.45) of ^18^F-pentixafor in our study is relatively lower than that of ^68^Ga-pentixafor (7.65), which might be explained by the differences between two nuclide-labeled radiotracers (18, 25, 27). Additionally, among semi-quantitative parameters, LAR emerged as the most effective indicator for ^18^F-pentixafor in our study, while LLR was identified as the best indicator for ^68^Ga-pentixafor in the study by Zheng et al. This correspondence may be substantiated by the comparison of tumor-to-organ ratios between ^18^F-AlF-NOTA-pentixafor and ^68^Ga-pentixafor as described by Poschenrieder et al (25).

The small size of nodules was considered to limit the subtype diagnosis of adrenal CT, and a few previous PET/CT studies (17, 20, 28) dispute the diagnostic disadvantage associated with nodules < 1 cm in diameter. Our study found that in lesions < 1 cm in diameter, the sensitivity of PET/CT visual analysis was as high as 72.73%. Additionally, our study indicated a positive correlation between SUVmax and lesion diameter in PA patients (r=0.351, P=0.001), a finding also reported in prior studies (20, 28). Given that previous studies have reported a negative correlation between adenoma size and CYP11B2 expression (29) and a positive correlation between the expression level of CXCR4 and CYP11B2, the expression density of CXCR4 in micro-APA should theoretically be higher than that in macro-APA. We deem the uncertainty in the relationship between SUV_max_ and nodule diameter may be attributed to the restricted spatial resolution of PET/CT imaging, which was also mentioned by prior research (30).

Our study showed that the positive predictive value (PPV) of the main positive lesions to diagnose UPA and the main cold lesions to diagnose NFA was 100%. However, in 15 warm lesions, 7 were proved UPA, 4 were proved BPA, and 4 were proved NFA. In previous studies, adrenal lesions with a similar uptake to contralateral and adjacent normal adrenal tissue were diagnosed as non-functional nodules by PET/CT (20, 28), which caused 5 missed cases out of 12 with a false negative rate of 41.66 in our study. This finding revealed that warm lesions were more likely to cause diagnostic ambiguity and be misdiagnosed. Therefore, PET/CT diagnosis in such lesions should be more careful, and multi-dimensional analyses (including semi-quantitative parameter analysis and nodule characteristic analysis) are necessary. In 20 patients who underwent AVS, the concordance rate of AVS and PET/CT visual diagnosis was 65.00%. Our study suggested that the adrenal gland with higher ^18^F-pentixafor uptake but no anatomical abnormalities might be misdiagnosed as dominant secretion side by PET/CT visual diagnosis. Therefore, it was unreliable to rely solely on radiotracer uptake for determining laterality, and anatomical findings should be taken into consideration as well.

There are several limitations to this study. First, it was a single-center study with a small sample size, especially in the AVS patients. Second, since there are only 4 BPA patients confirmed by AVS in our study, the results may be biased due to the uneven distribution of the sample size. More confirmed BPA samples are required. Third, the sample size of lesions < 1 cm in diameter was small. Fourth, resected adrenal lesions were not routinely sent for CYP11B2 staining in our hospital and the pathological diagnosis of adrenal lesions was based solely on morphology with hematoxylin-eosin (HE) staining.

## 5 Conclusion

In summary, this study demonstrates that CXCR4-targeted ^18^F-AlF-NOTA-pentixafor PET/CT is a valuable noninvasive tool to diagnose UPA with high sensitivity and specificity and to guide further treatment options. Adrenal lesions with a similar uptake to contralateral and adjacent normal adrenal tissue are more likely to cause diagnostic ambiguity and misdiagnosis, requiring more attention from nuclear medicine physicians.

## Data Availability

The raw data supporting the conclusions of this article will be made available by the authors, without undue reservation.
