# Corrigendum: The value of targeted CXCR4 ^18^F-AlF-NOTA-pentixafor PET/CT for subtyping primary aldosteronism

**DOI:** 10.3389/fendo.2025.1593408

**Published:** 2025-04-15

**Authors:** Yushi Peng, Fangansheng Chen, Rui Yao, Junping Lan, Yinuo Fu, Kaifeng Ye, Zhiqiang Wang, Qianxiu Zhao, Xiaowei Ji, Kang Xia, Guoqing Zhu, Kewen Zheng, Xuemei Gu, Kun Tang

**Affiliations:** ^1^ Department of Nuclear Medicine, The First Affiliated Hospital of Wenzhou Medical University, Wenzhou, China; ^2^ Department of Radiology, The First Affiliated Hospital of Wenzhou Medical University, Wenzhou, China; ^3^ The First School of Clinical Medicine, Wenzhou Medical University, Wenzhou, China; ^4^ Department of Endocrinology, The First Affiliated Hospital of Wenzhou Medical University, Wenzhou, China; ^5^ Department of Interventional Radiology, The First Affiliated Hospital of Wenzhou Medical University, Wenzhou, China; ^6^ Department of Urology, The First Affiliated Hospital of Wenzhou Medical University, Wenzhou, China; ^7^ Key Laboratory of Intelligent Treatment and Life Support for Critical Diseases of Zhejiang Province, Wenzhou, China; ^8^ Key Laboratory of Novel Nuclide Technologies on Precision Diagnosis and Treatment & Clinical Transformation of Wenzhou City, Wenzhou, China

**Keywords:** primary aldosteronism, CXCR4, 18 F-pentixafor, PET/CT, subtyping, treatment

In the published article, there was an error in **Figure 7** as published. We mistakenly exported and uploaded this figure. The corrected **Figure 7** and its caption appear below.

**Figure 7:**
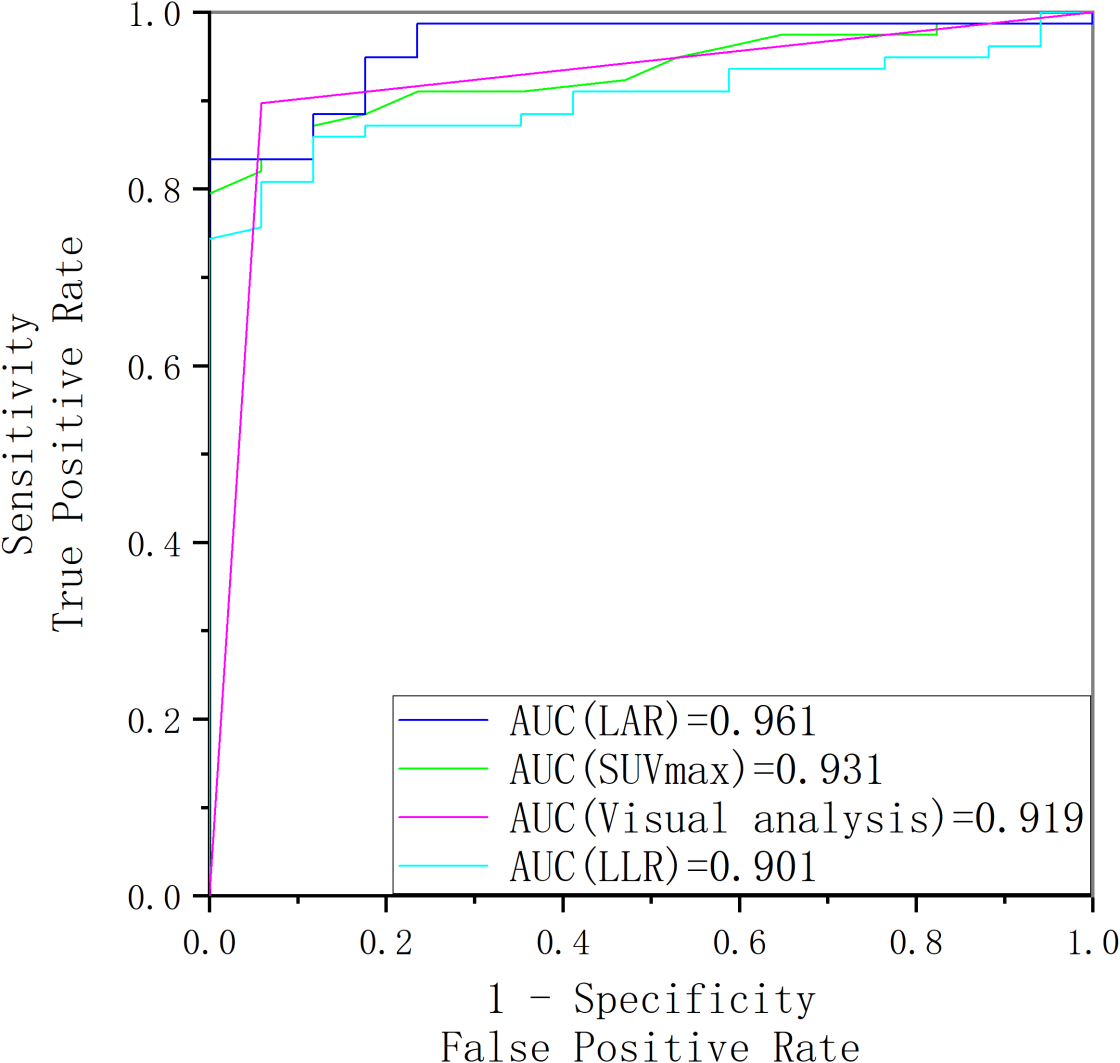
The receiver operating characteristic curve of SUV_max_, LAR, LLR and visual analysis for identifying surgically eligible lesions and surgically ineligible lesions.

In the published article, there was an error in section 2. Materials and Methods, *Adrenal Venous Sampling Interpretation*, Paragraph 1. The following sentence was removed.

“If the ratio of aldosterone/cortisol in the high-side adrenal vein blood to the low-side adrenal vein blood is 4 or greater, it indicates increased aldosterone secretion from one side of the adrenal gland, which is diagnosed as APA.”

In the published article, there was an error in section 3. Results, *Diagnostic Accuracy of 18F-AlF-NOTA-pentixafor PET/CT for surgically eligible lesions*, Paragraph 1. The following sentence was removed.

“Among enrolled patients, 2 UPA patients exhibited 2 hot nodules in the ipsilateral adrenal gland, 2 UPA patients exhibited a cold lesion contralateral to the UPA lesion, and 3 NFA patients exhibited bilateral nodules. The remaining 81 patients displayed unilateral adrenal gland lesions on CT scans.”

The authors apologize for these errors and state that this does not change the scientific conclusions of the article in any way. The original article has been updated.

