# Supplementary material for: The value of targeted CXCR4 18F-AlF-NOTA-pentixafor PET/CT for subtyping primary aldosteronism
Source: Front Endocrinol (Lausanne). 2025 Feb 27;16:1533295. doi: 10.3389/fendo.2025.1533295 (PMC11903271; doi:10.3389/fendo.2025.1533295)
Supplement: Supplementary file 1 [file Table1.docx]

**Supplement 1** Clinical Characteristics of Included Patients with main lesions < 1 cm and ≥ 1 cm in diameter

| **Characteristic** | **Patients, Median (P_25_, P_75_)** | | **P** |
| --- | --- | --- | --- |
|  | **< 1 cm** | **≥ 1 cm** |  |
| Age, y, mean±SD | 58.50±15.04 | 52.90±8.11 | .332 |
| Gender(male/female) | 5/6 | 40/37 | .755 |
| BMI, mean±SD | 24.70±3.09 | 25.33±3.28 | .571 |
| Number of hypertension | 11/11 | 74/77 | 1.000 |
| Duration of hypertension, y | 10.00 (8.00,20.00) | 7.00 (2.00,15.00) | .282 |
| Systolic pressure, mmHg | 162 (153,180) | 161(149,175) | .929 |
| Diastolic pressure, mmHg | 100(88,105) | 96(89,108) | .793 |
| Number of hypokalemia | 9/11 | 57/77 | .852 |
| Serum potassium, mmol/L | 3.19(3.01,3.41) | 3.20 (2.86, 3.55) | .655 |
| Plasma aldosterone concentration, pg/mL | 312.61(153.44, 371.47) | 293.03(193.68, 521.76) | .583 |
| ARR, pg·mL^−1^/ pg·mL^−1^ | 130.03(90.26,199.93) | 139.77(58.62, 393.96) | .557 |
