# Supplementary material for: The value of targeted CXCR4 18F-AlF-NOTA-pentixafor PET/CT for subtyping primary aldosteronism
Source: Front Endocrinol (Lausanne). 2025 Feb 27;16:1533295. doi: 10.3389/fendo.2025.1533295 (PMC11903271; doi:10.3389/fendo.2025.1533295)
Supplement: Supplementary file 2 [file Table2.docx]

**Supplement 2** The Clinical Characteristics of 7 patients with a disagreement between PET/CT visual diagnosis and AVS.

| **Patient** | **Age, y** | **Sex** | **Serum potassium, mmol/L** | **PAC, pg/mL** | **PRC, pg/mL** | **ARR, pg·mL^−1^/ pg·mL^−1^** | **Lesion diameter, mm** | **Characteristic of nodule** | **Lesion SUV_max_** | **LI based on SUV_max_** | **LI based on AVS** | **PAC post-SSIT, pg·mL^−1^** | **PAC post- CCT, pg·mL^−1^** | **Cortisol post-ODST, nmol·L^−1^** |
| --- | --- | --- | --- | --- | --- | --- | --- | --- | --- | --- | --- | --- | --- | --- |
| No. 7 | 76 | F | 4.40 | 208.15 | 1.02 | 204.07 | 8 | Unilateral single warm nodule | 3.1 | 1.15 | 5.26 | 189.65 | 111.09 | 24.11 |
| No. 11 | 58 | M | 3.70 | 733.34 | 0.5 | 14666.8 | 16 | Bilateral negative nodules  (left warm nodule and right cold nodule) | 3.5 | 1.13 | 3.50 | 110.98 | 117.27 | 29.39 |
| No. 13 | 54 | M | 3.55 | 252.32 | 5.26 | 47.97 | 7 | Left multifocal warm nodules and right adrenal ramus medialis with increased uptake but no anatomical abnormalities | 3.9 | 1.38 | 12.34 | 223.43 | 221.25 | 22.68 |
| No. 21 | 56 | M | 3.49 | 255.12 | 12.67 | 20.14 | 10 | Unilateral single warm nodule | 4.2 | 1.27 | 6.14 | 139.81 | 154.15 | 17.01 |
| No. 24 | 67 | M | 2.76 | 1822 | 11.51 | 158.3 | 13 | Bilateral warm nodules | 2.7 | 1.04 | 28.00 | 529.54 | 340.51 | 21.89 |
| No.68 | 52 | M | 2.86 | 322.3 | 0.79 | 407.97 | 24 | Bilateral warm nodules | 3.4 | 1.13 | 2.00 | 660.43 | 483.73 | 38.00 |
| No.81 | 47 | M | 2.78 | 632.48 | 7.66 | 82.57 | 14 | Left multifocal hot nodules and right single warm nodule (right adrenal ramus lateral nodular thickening with sightly higher uptake compared to adjacent normal adrenal tissue) | 11.8 | 2.88 | 5.83 | 338.44 | 262.17 | 33.12 |

PAC: plasma aldosterone concentration; PRC: plasma renin concentration; ARR: Ratio of PAC to PRC; SSIT: seated saline infusion test; CCT: captopril challenge test; ODST: overnight dexamethasone suppression test, *Serum potassium was the lowest level in the medical history before treatment of hypokalemia.
